# Combined Use of Modal Analysis and Machine Learning for Materials Classification

**DOI:** 10.3390/ma14154270

**Published:** 2021-07-30

**Authors:** Mohamed Abdelkader, Muhammad Tayyab Noman, Nesrine Amor, Michal Petru, Aamir Mahmood

**Affiliations:** 1 Department of Advanced Materials, Institute for Nanomaterials, Advanced Technologies and Innovation (CXI), Technical University of Liberec, 461 17 Liberec, Czech Republic; mohamed.fawzy@mena.vt.edu; 2 Department of Mechanical and Materials Engineering, Vilnius Gediminas Technical University, 10221 Vilnius, Lithuania; 3 Department of Nanoengineering, Center for Physical Sciences and Technology (FTMC), 02300 Vilnius, Lithuania; 4 Department of Machinery Construction, Institute for Nanomaterials, Advanced Technologies and Innovation (CXI), Technical University of Liberec, 461 17 Liberec, Czech Republic; nesrine.amor@tul.cz (N.A.); michal.petru@tul.cz (M.P.); 5 Department of Material Engineering, Faculty of Textile Engineering, Technical University of Liberec, 461 17 Liberec, Czech Republic; aamir.mahmood@tul.cz

**Keywords:** isotropic, anisotropic, orthotropic, modal analysis, resonance frequency, mode shapes

## Abstract

The present study deals with modal work that is a type of framework for structural dynamic testing of linear structures. Modal analysis is a powerful tool that works on the modal parameters to ensure the safety of materials and eliminate the failure possibilities. The concept of classification through this study is validated for isotropic and orthotropic materials, reaching up to a 100% accuracy when deploying the machine learning approach between the mode number and the associated frequency of the interrelated variables that were extracted from modal analysis performed by ANSYS. This study shows a new classification method dependent only on the knowledge of resonance frequency of a specific material and opens new directions for future developments to create a single device that can identify and classify different engineering materials.

## 1. Introduction

Materials play an essential role in making products functional, versatile and boosting their performance to a significant level. Therefore, understanding the materials’ behavior is crucial in designing reliable products [1]. Materials can be classified according to multiple perspectives; one of these perspectives is the identification of material properties according to different mechanical response parameters. Materials are categorized mainly into two categories according to their mechanical properties: isotropic and anisotropic. Isotropic materials have the same properties (mechanical, physical, electrical, etc.) when measured from any direction, i.e., isotropic materials are direction independent, while anisotropic materials have direction-dependent properties. Anisotropic materials have their properties as a function of measurement orientation or angle. Orthotropic materials are a special type of anisotropic material. As a special class of anisotropic material, orthotropic materials have three mutually orthogonal planes of elastic symmetry in which their characteristics remain the same [2]. Structures can experience failure when they resonate at certain frequencies. Modal analysis is a method of extracting modal parameters (natural frequencies, damping loss factors and modal constants) from obtained vibrational data. Modal analysis is considered as one of the non-destructive methods to evaluate material properties. Modal analysis can be analyzed in time domain or frequency domain. The data obtained by modal analysis are fed to machine learning algorithms in order to learn and predict new or unknown variables or values [3]. Machine learning (ML), a subcategory of artificial intelligence, is the ability of computerized systems to autonomously find solutions to problems by recognizing repeated patterns in datasets. In other words, ML is an approach to solve new problem inputs based on experience from previous inputs [4]. Before exploring different approaches to apply ML or deep learning (DL) algorithms, it is necessary to differentiate the types of datasets that may exist in the problem. Datasets are categorized into three types: training datasets, test datasets and validation datasets. Training datasets are those sets that have the data and their tags with the purpose of training the model to learn how to classify new inputs of data [5]. By applying ML and DL approaches, data trends are explored by Python3 on the Jupyter Notebook interface.

In recent years, researchers have worked with modal analysis tools and applied them under multiple applications particularly in mechanical engineering, civil engineering, aerospace engineering and textile composites [6,7,8,9,10,11,12,13,14,15]. Anastasopoulos et al. worked with modal analysis for steel bridge analysis, evaluating the influence of temperature and retrofitting, and explained that the dynamic strain and bridge properties were automatically detected and analyzed through this approach [16]. In another identification of shear compression, Carpine et al. used modal analysis on a masonry wall equipped with accelerometers under ambient excitations and investigated the effects of closed frequencies, reporting that modes with natural frequencies can enhance the controlling process of heavy noise [17]. Fang and Zhang used the modal analysis method for the improvement of vibro-acoustic problems under the hybrid finite element/statistical energy method. They used mode shapes to minimize the order of acoustic elements modelled by the finite element method. Their results explain that the proposed method has significant advantages over the traditional finite element method in computational efficiency [18]. In literature, several research groups investigated the materials’ properties via modal analysis with an extension to establish and study the correlations and relationships between material properties and the results of modal analysis [19,20,21,22,23]. Classification is the process of categorizing the data into specific classes [24,25,26].

In the current study, modal analysis is used as a simulation tool performed by ANSYS software (Ansys, Inc., Canonsburg, PA, USA) (accessed on 02 July 2021). Generally, the first 13 modes are considered to be sufficient in engineering analysis, however, in our study, the first 100 modes were obtained to provide enough data for training and an accuracy check of the models. The main aim of this study is to provide and elucidate a concrete classification method based only on the obtained frequencies from modal analysis simulation, eliminating the need of any additional method to perform such a classification. We propose a new method that can be considered as a supervised machine learning approach. The first part of the study discusses the application of ML and DL algorithms on the data obtained from stainless steel and magnesium alloy and further validates which one is better for a high accuracy classification scheme. In the second step, the trained pattern is applied to the orthotropic materials epoxy carbon woven (230 GPa) wet and epoxy E-glass UD and their results obtained. It was noticed that the better approach was the ML approach compared to the Keras model, which is a deep learning approach. In ML, different algorithms are explored including logistic regression, decision tree classification and more. To get the data ready for the ML model, the 100 samples were divided into two parts: 80% of the data for the training purposes and the other 20% of the data for the testing of the model. The core point of this research is the establishment of a classification method dependent only on the resonance frequencies, compared to previous work that depended on the frequency domain analysis and more than one variable to be able to make the classification.

## 2. Materials and Methods

### 2.1. Materials

Three types of isotropic materials, i.e., magnesium, steel structure and copper alloy, and two types of anisotropic (orthotropic) materials, i.e., epoxy carbon woven (230 GPa) wet and epoxy E-glass UD, were used in this study. All materials used have rectangular shape with dimensions of 250 mm in length, 125 mm in width and 5 mm in thickness. All materials properties are the default properties that ANSYS sets for each material included in the ANSYS materials library.

### 2.2. Modal Analysis

The prediction of the resonance frequency of a structure is extremely beneficial so that one can grasp fundamental dynamic behavior and eliminate the possibility of resonance while being in service. The main applications of modal analysis are computation of the critical speed of rotating machines and noise reduction through vibration isolation of the machine parts. The general form of modal analysis is given in Equation (1).

(1) $$\left[M\right]\left[U^{\prime \prime }\right]+\left[C\right]\left[U^{\prime }\right]+\left[K\right]\left[U\right]=\left[F\right]$$

In the above equation, *M* represents the mass matrix, $U^{\prime \prime }$ (acceleration) is the second derivative of displacement, *C* represents the damping matrix, $U^{\prime }$ is the velocity, *K* shows the stiffness matrix, *U* is the displacement and *F* is the external force vector whose value is equal to zero.

**Figure 1:**
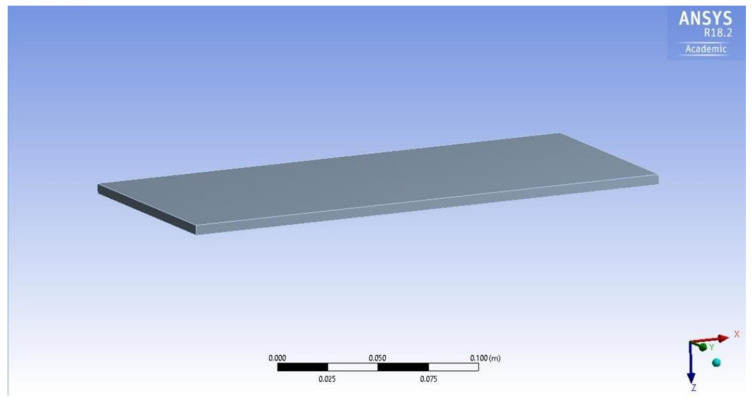
The studied plate with dimensions of 250 mm in length, 125 mm in width and 5 mm in thickness, using ANSYS.

ANSYS 18.2 was used for modal analysis throughout the study. A simple apparatus of the long rectangular plate is illustrated in Figure 1.

**Figure 2:**
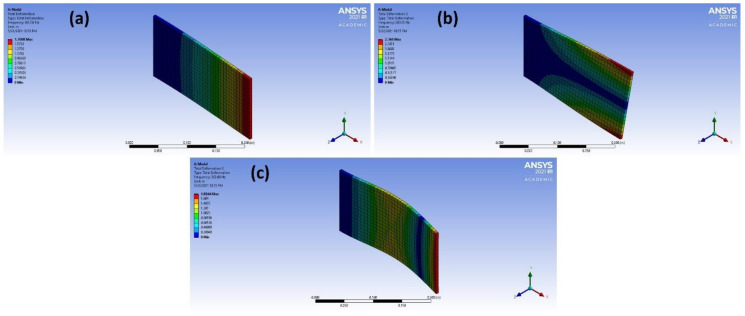
(**a**–**c**) Overview of different mode shapes obtained of the copper plate after the processing of data.

The plate was fixed in the z direction from one end. To check the mesh quality, the mesh metrics of the model were checked. The minimum orthogonal mesh quality was 1 and the skewness was 1.306 e^−010^ <<< 0.95 which is assuring that the mesh is suitable and of a high quality for further calculations. Modal analysis was performed with the same plate, changing the material each time, for collecting the results in order to find general relations or trends that govern the behavior of these materials using ML. In addition, mode shapes were obtained that elucidate the displacement under the resonance frequency. The first three mode shapes of copper plate are illustrated in Figure 2. The resonance frequencies were extracted and later read by Python (Python Software Foundation, Wilmington, DE, USA) (accessed on 2 July 2021) in order to process the data.

#### Kirchhoff Thin Plate Model

**Figure 3:**
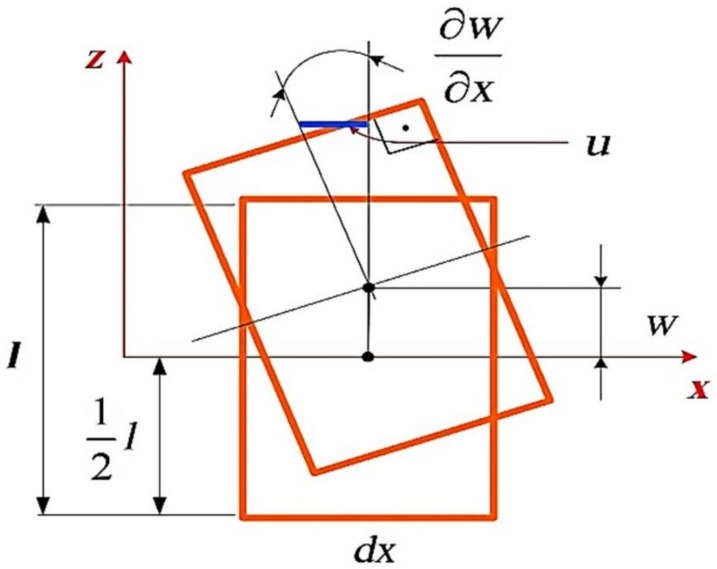
The explanation of the Kirchhoff-Love plate model. Where 1 is the plate thickness, u is the plate displacement amplitude, w is the difference between centers of deformed and non-deformed plate, δw/δx is the strain due to the applied force on the element and dx is an infinitesimal element in the x-axis.

The effects of changing plate sides on modal analysis were followed by the Kirchhoff–Love theory of plates. This method is a two-dimensional (2D) mathematical model that is used to find the stresses and deformations in thin plates exposed to forces and moments. This model assumes that a mid-surface plane can be used to represent a three-dimensional (3D) plate in 2D form. The below equation (2) shows the conditions for the Kirchhoff thin plate (where l is the plate thickness and L is the plate length) and Figure 3 shows the model.

(2) l <0.1 L 

### 2.3. Data Processing

In this study, materials classification or categorization is identified based on the modal analysis simulation results, after which a supervised ML or DL scheme is used. Supervised machine learning is a type of ML approach that utilizes a known dataset, known as the training dataset, to make predictions. The training dataset includes input variables denoted as (“X”) and output or response variables denoted as (“Y”). From these variables, a supervised learning algorithm creates a model that can predict the output variables (“Y”) for an unknown dataset (the testing dataset) that can be used to check the accuracy of the model. The difference between ML and DL is that DL can conduct the features extraction and classification together whereas ML cannot. ML and DL approaches can be broadly categorized into supervised and unsupervised learning. Supervised learning assigns the accurate classifications to a dataset and uses them to train the algorithm. Alternatively, in unsupervised DL, the algorithm can learn hidden and inherent patterns within a dataset by self-organizing [27].

#### 2.3.1. Deep Learning Approach

When building a neural network, regardless of the interfacing package used for the model, all networks follow the same general scheme. Figure 3 shows the general flow design of a neural network: it starts with feeding the datasets and ends with testing the trained model to predict new data feeds.

A Python structure for the Keras model was created for a comparative analysis of the obtained results. DL always uses large datasets, as larger datasets can achieve more accurate predictions. Our model can predict new data feeds efficiently. The first attempted approach was picking up and tagging the two analyzed materials with ANSYS, stainless steel and magnesium alloy. Stainless steel was tagged with “0” and magnesium alloy was tagged with “1”. The input to Keras was two dimensional (two columns of “X”), where the first was the mode number and the second was the natural frequency of the selected mode. The output of the training dataset for Keras was the tag, being “0” or “1”. The same data pre-processing approach was applied to the orthotropic materials and the results were obtained; epoxy carbon woven (230 GPa) wet was tagged with “0” and epoxy E-glass UD was tagged with “1”. The Keras data was divided into two parts, the training set and the test set. It was divided in a manner so that 80 samples out of 100 for each material would be in the training set and the rest (20 samples each) would be for the test set to evaluate the accuracy of the model.

#### 2.3.2. Machine Learning Approach

Different ML methods, such as logistic regression, decision tree classification, K-nearest neighbors, linear discriminant analysis, Gaussian naive Bayes and support vector machine, were explored in this study. Logistic regression is a suitable regression method when dealing with data that have the dependent variable in binary form. A decision tree is a probabilistic classifier that uses data observations to predict the classification process output. K-nearest neighbors (KNN) is one of the fundamental classification methods and should be used in cases of no prior information on the data distribution. Linear discriminative classification/analysis (LDA) is used in case of unequal frequencies of occurrence in the dataset with some randomness. LDA is a well-known technique in data pattern recognition and has been used in speech recognition. Bayes’ theorem is used to determine the conditional probability of parameter values through combining expectations based on previously known data with information from available data [28,29,30]. A support vector machine (SVM) is used for nonlinear problems and higher dimensional spaces.

To increase the model possibility for higher prediction probabilities, another column was added to the input data of the model, using the combined linear regression and ML method by constructing the “|𝑚 × 𝑏|” term for each material where “m” and “b” are the linear regression variables, in other words, applying linear regression separately to each of the material results. Linear regression is one of the basic tools in statistics to correlate between different variables for a set of observations. Applications of linear regression can vary from simple tasks, such as simple line fitting, to a much more complex task, such as facial recognition.

#### 2.3.3. Model Evaluation

A data-driven model aims to reach a good performance not only on current data but also on new or unknown data. Generally, the evolution of degree of accuracy or error percentage of models can be done by different computational metrics and based on these results, the best model can be selected. Testing data are needed to test the model capability on a new dataset: with the test error obtained, the testing data can be taken as an indication of the general error. When there is only one dataset containing “*i*” samples, we can partition *D* into a training dataset S and a testing dataset T for training and testing, respectively, using different evaluation methods. The equation (3) shows the dataset pairs where *D* represents the data set, *X* represents the input and *Y* represents the output.

(3) $$D=\left\{\left(x_{1},y_{1}\right),\left(x_{2},y_{2}\right),\ldots ,\;\left(x_{i},y_{i}\right)\right\}$$

There are different methods to measure the model accuracy and determine the error percentage. Some of the main measures are the mean absolute percentage error (*MAPE*), the root mean square error (*RMSE*) and the correlation coefficient ($R^{2}$), which are all used to evaluate models applied to measure the accuracy of regression or classification problems. Equations (4)–(6) show three famous accuracy metrics in machine learning.

(4) $$MAPE=\frac{1}{n}\sum_{i=1}^{n}\frac{\left|{y^{\prime }}_{i}-y_{i}\right|}{y_{i}}$$

(5) $$RMSE=\sqrt{\frac{1}{n}\sum_{i=1}^{n}{\left({y^{\prime }}_{i}-y_{i}\right)}^{2}}$$

(6) $$R^{2}=\frac{\left[\sum_{i=1}^{n}\left(y_{i}-\bar{y}\right){\left({y^{\prime }}_{i}-{\bar{y}}^{\prime }\right)}^{2}\right]}{\sum_{i=1}^{n}{\left(y_{i}-\bar{y}\right)}^{2}.\sum_{i=1}^{n}{\left({y^{\prime }}_{i}-{\bar{y}}^{\prime }\right)}^{2}}$$

In these equations, $y_{i}$ and ${y^{\prime }}_{i}$ represent an original value and its corresponding predicted value, respectively, while $\bar{y}$ and ${\bar{y}}^{\prime }$ are the averages of the original and predicted values, respectively.

#### 2.3.4. Data Processing and Analysis

**Figure 4:**
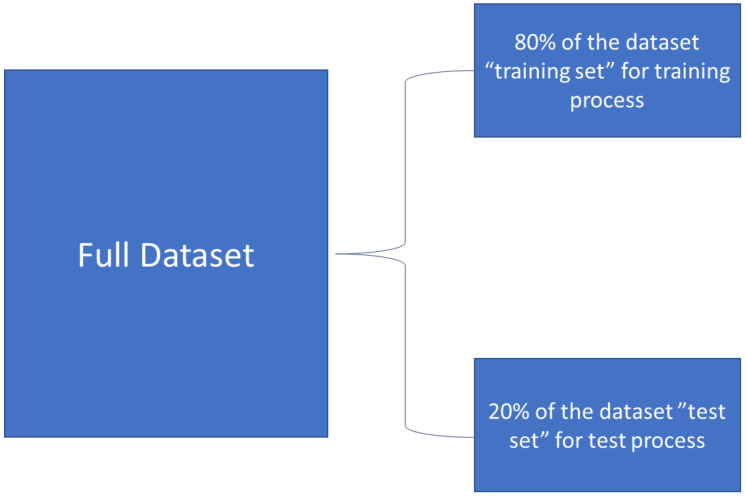
Extracted dataset separation into training set and test set.

After obtaining the data from the ANSYS simulation, the subsequent task was to organize the “data pre-processing” data. To get the data ready for the ML model, the 100 samples were divided into two parts: 80% of the data for the training purposes and the other 20% of the data for the testing of the model. Figure 4 shows the concept of dataset separation into the training set and test set. For exploring data trends and applying machine learning and deep learning approaches, Python3 was used on the Jupyter Notebook (Open-source platform, Project Jupyter, https://jupyter.org/) (accessed on 2 July 2021) interface.

**Figure 5:**
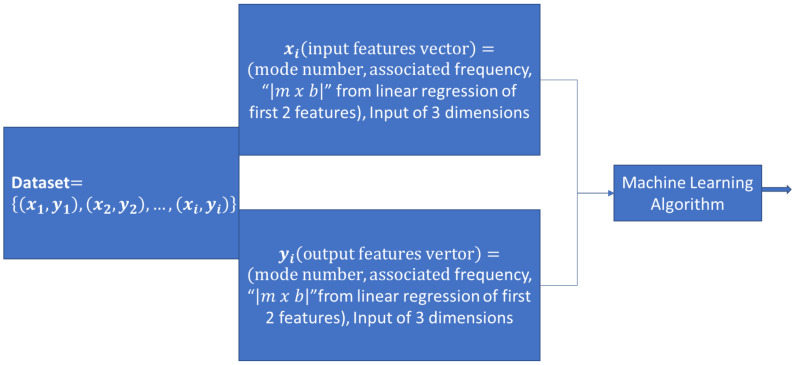
Dataset pre-processing and feeding into machine learning model.

Before exploring different approaches to apply machine learning [ML] or deep learning [DL] approaches, a discrimination between the types of data we may have in the problem must be completed first. Datasets can be categorized into 3 types: training datasets, test datasets and validation datasets. Training datasets are sets that have the data and their tags with the purpose of training the model to learn how to classify new inputs of data. The dataset was organized in a CSV Excel file with the “X” input portion of the data having a dimension of “3” and the “Y” corresponding output with a dimension of “1”. The dimensions of the input are the mode number, the associated frequency and the “|𝑚 × 𝑏|” term which is extracted from the linear regression between the mode number and its associated frequencies. Linear regression is one of the basic tools in statistics to correlate between different variables for a set of observations. Applications of linear regression can vary from simple tasks, such as simple line fitting, to a much more complex task, such as classification and pattern recognition [31,32,33]. To increase the model possibility for higher prediction probabilities, another column was added to the input data of the model, using the combined linear regression and ML method by constructing the “|𝑚 × 𝑏|” term for each material where “m” and “b” are the linear regression variables, in other words, applying linear regression separately to each of the material results. Figure 5 shows the data preprocessing step before feeding the data into the machine learning algorithm and checking the model accuracy.

## 3. Results and Discussions

The first three frequencies for the different materials investigated through this study are shown in Table 1. Stainless steel and magnesium were explored to validate the model applicability on isotropic materials, while epoxy carbon woven (230 GPa) wet and epoxy E-glass UD were used to validate the model applicability on orthotropic materials.

After applying the Keras model for both isotropic and orthotropic studied materials, the achieved accuracy did not exceed 50% even with the combined linear regression approach, which proved that the Keras model was not suitable for this study. Alternating the model parameters did not lead to any improvement in that method, which proves that the Keras model was not a suitable DL approach for this study. However, other DL models or neural networks may show promising results.

### 3.1. Isotropic Materials

To summarize the obtained results, Table 2 shows the obtained results of accuracies for different ML algorithms. From the table, we conclude that the decision tree and SVM are the best methods for training accuracy, however they lack accuracy in the test sets compared to the other methods. In this section, the |𝑚 × 𝑏| relationship is added to the “X” input as an additional input dimension to the datasets. Table 3 shows accuracy values for different machine learning algorithms after applying the combined approach. The “|𝑚 × 𝑏|” term equaled 4515 for stainless steel and 1663 for magnesium.

**Figure 6:**
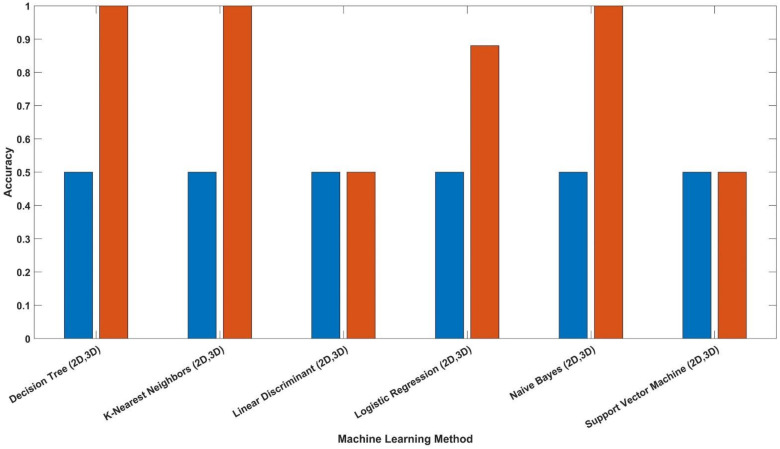
Isotropic test set accuracy results for 2D and 3D input to the ML model using different ML approaches.

**Figure 7:**
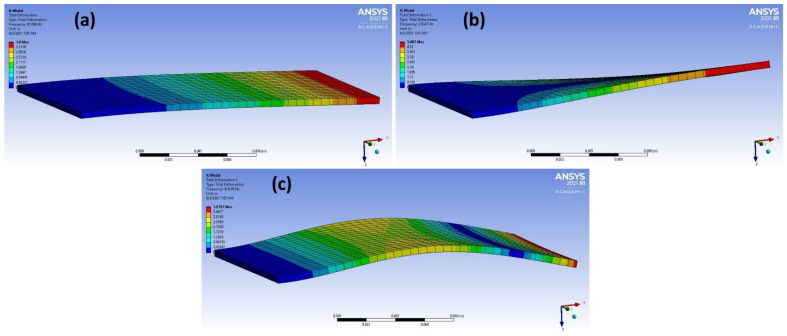
(**a**–**c**) 3 different mode shapes of magnesium alloy after data processing.

**Figure 8:**
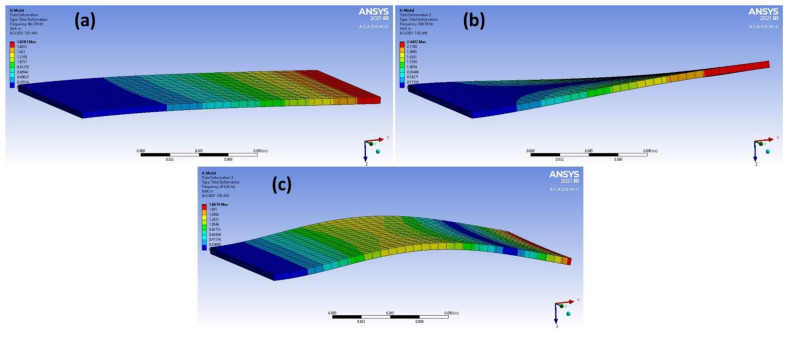
(**a**–**c**) 3 different mode shapes of steel after data processing.

From the results in Table 3, it was found that adding the new dimensions to input vectors that were based on the term “|𝑚 × 𝑏|” obtained from linear regression strongly aided in increasing the accuracy of the models of different methods. The accuracy of predicting the correct material classification that corresponds to the input frequency increased the accuracy of the test set from ~ 50 to 100% in the logistic regression, decision tree, KNN and naïve Bayes methods. Figure 6 shows the accuracy values for the different ML models after adding the |𝑚 × 𝑏| dimension. The mode shapes of the isotropic materials (magnesium and steel structures) after processing the data are shown in Figure 7 and Figure 8, respectively. The mode shapes illustrate the deformation displacement of the body responding to the resonance frequencies. The induced deformation increased as the plate moved away from the fixed face. In simple terms, when a body oscillates at natural or resonance frequency, it may lead to total failure of the body. The deformation of the body increased as the movement along the body moved away from the fixed face of the body.

**Figure 9:**
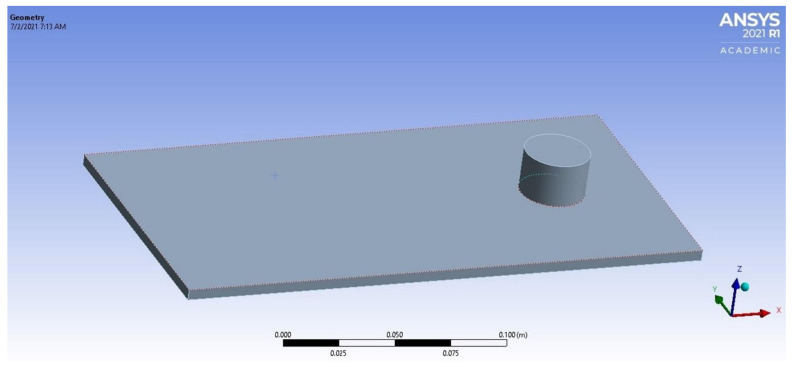
The modified shape of the plate.

**Figure 10:**
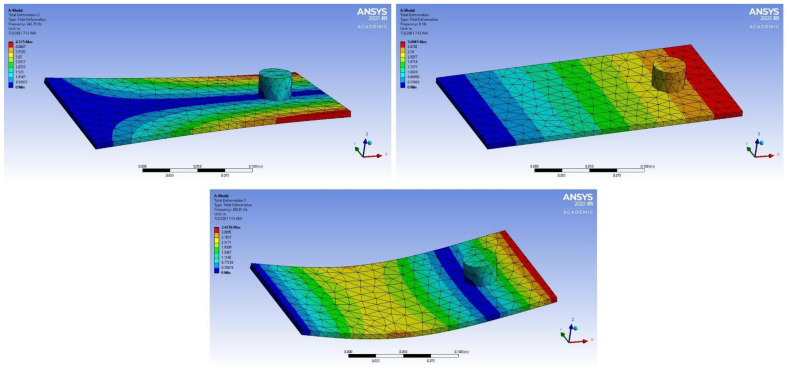
Mode shapes of magnesium using the modified plate.

In addition, it should be noted that this study is more focused on a data approach in which the shape of the studied part is not a factor that affects the validity of the study as the proposed method only deals with numbers/extracted data of the modal analysis results. The simple shape that has been investigated in this study is used to verify the concept, but in general, the algorithm efficiency should be independent of the studied shape. In order to testify the validity of our algorithm in that the algorithm is independent of the sample shape, the shape has been modified through adding a cylindrical part to the plate as shown in Figure 9. However, Figure 10 shows the first three mode shapes using the modified plate with magnesium as the material of the shape. It was observed from the results that the algorithm maintains reliability through the replacement of shapes.

### 3.2. Orthotropic Materials

As the combined linear regression approach proved its efficiency in increasing the model accuracy up to 100%, it was applied directly to the orthotopic materials. The “|𝑚 × 𝑏|” term equaled 279,739 for epoxy carbon woven (230 GPa) wet and 43,094 for epoxy E-glass UD. Table 4 shows accuracy values for different machine learning algorithms after applying the combined approach on the orthotropic materials data. It was observed that the best fitted models were decision tree, K-nearest neighbors and naïve Bayes as they scored an accuracy of 100%, compared to the logistic regression and support vector machine algorithms.

In order to compare the isotropic and anisotropic materials’ samples using a 3D test dataset, the accuracy of data for both cases of isotropic and anisotropic materials are given in Table 5. It was observed (Table 5) that the methods of decision tree, K-nearest neighbors and naive Bayes gave the maximum possible accuracy, meaning that these three methods are suitable candidates for further investigations.

**Figure 11:**
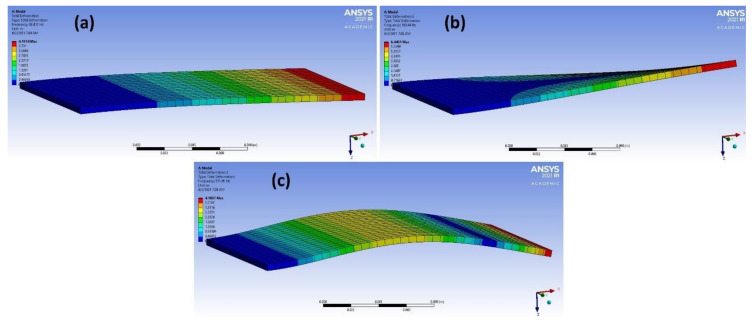
(**a**–**c**) 3 different mode shapes of epoxy carbon woven (230 GPa) wet after data processing.

**Figure 12:**
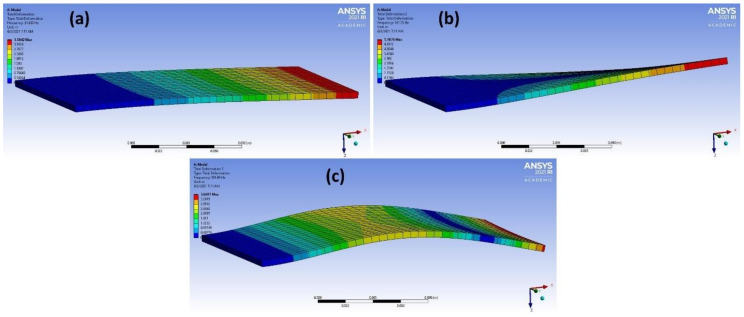
(**a**–**c**) 3 different mode shapes of epoxy E-glass UD after data processing.

**Figure 13:**
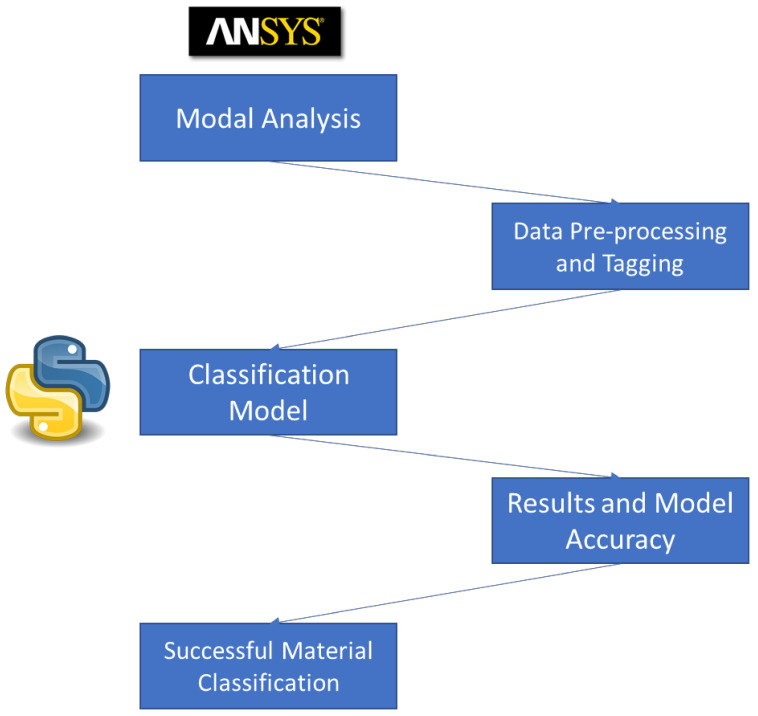
Overall summary and procedure of using ML and DL in engineering materials classification.

The mode shapes of the orthotropic materials epoxy carbon woven (230 GPa) wet and epoxy E-glass UD, after processing the data, are shown in Figure 11 and Figure 12, respectively. To summarize the results of this study by using ML and DL approaches in order to classify engineering materials based on their modal response, Figure 13 elucidates the study summary starting from the modal analysis in ANSYS, getting the dataset, data pre-processing, applying different machine learning and deep learning algorithms and ending with checking the model validity and its accuracy.

## 4. Conclusions

- The study demonstrated the ability to classify engineering materials, including isotropic and orthotropic materials, by applying ML algorithms on the modal analysis results. The proposed ML approaches could reach an accuracy of 100% when interrelations were created between the inputs to the ML algorithms (combined linear regression approach in this study).
- The Keras model was not suitable for this study as it showed 50% accuracy when compared to the ML approaches. The study validates the classification applicability based on the resonance frequency information, which may broaden the horizon of further applications such as a device that can classify the materials based on their modal analysis.
- The study showed a novel method of using the extracted data from modal analysis to accurately classify and identify the engineering materials as well as validate the efficiency of using induced interrelations between the mode number and its corresponding resonance frequency to increase the accuracy of the proposed machine learning methods.
- The study validated the classification applicability based on the resonance frequency information, which may broaden the horizon of further applications such as a device that can classify the materials based on their modal analysis. A further study could include other modal analysis parameters, such as damping ratios using experimental data as it will be by default assumed to be zero in ANSYS.
- Potential future studies can study other DL approaches and the deployment of neural networks that could achieve promising classification studies. Further extensions and relations can be established for detailed material properties identification through deploying the concept of ML and DL into the field of mechanical engineering, which would further confirm the modern concept of science integration.
- The results of this study can boost non-destructive materials characterizations and analysis methods in general, not just the explored modal analysis example, as ML and DL deal mainly with data regardless of the acquiring method.

## Figures and Tables

**Table 1:** Results of ANSYS modal analysis for the studied materials.

Mode Number	Frequency for the Studied Materials [Hz]			
	Stainless Steel	Magnesium	Epoxy Carbon Woven (230 GPa) Wet	Epoxy E-Glass UD
1	66.376	67.086	82.419	61.665
2	280.78	278.47	169.44	161.35
3	412.82	416.48	511.09	383.84

**Table 2:** Accuracy values for different machine learning algorithms.

Accuracy	Logistic Regression	Decision Tree	K-Nearest Neighbors	Linear Discriminant	Naive Bayes	Support Vector Machine
Training set	0.55	1.00	0.51	0.56	0.51	1.00
Test set	0.50	0.50	0.50	0.50	0.50	0.50

**Table 3:** Accuracy values for different machine learning algorithms with the combined linear regression approach applied on the isotropic materials’ results.

Accuracy	Logistic Regression	Decision Tree	K-Nearest Neighbors	Linear Discriminant	Naive Bayes	Support Vector Machine
Training set	1.00	1.00	1.00	0.56	1.00	1.00
Test set	0.88	1.00	1.00	0.50	1.00	0.50

**Table 4:** Accuracy values for different machine learning algorithms with the combined linear regression approach applied on orthotropic materials’ results.

Accuracy	Logistic Regression	Decision Tree	K-Nearest Neighbors	Linear Discriminant	Naive Bayes	Support Vector Machine
Training set	0.88	1.00	1.00	0.96	1.00	1.00
Test set	0.68	1.00	1.00	1.00	1.00	0.50

**Table 5:** Accuracy values for different machine learning algorithms applied on the 3D test datasets for isotropic and orthotropic materials.

Accuracy	Logistic Regression	Decision Tree	K-Nearest Neighbors	Linear Discriminant	Naive Bayes	Support Vector Machine
Isotropic	0.88	1.00	1.00	0.50	1.00	0.50
Orthotropic	0.68	1.00	1.00	1.00	1.00	0.50

## Data Availability

Not applicable.
